# Hernia. II

**Published:** 1895-03-09

**Authors:** 


### PROGRESS IN SURGERY.

### HERNIA.—II.

(*Continued from p. 367.*)

**Latent Hernia.**—Mr. Bennett calls attention to deceptive abdominal pains in latent hernia, especially with reference to the surgical aspect of certain cases of so-called indigestion.[1] He relates several very interesting cases in which attacks of abdominal pain, like colic, supposed to be due to indigestion or perityphitis, have really been caused by traction on a piece of omentum adherent in a very small hernia, which had not been discovered. The patients were all cured by radical cure of the hernia. Another case is also recorded where the same kind of abdominal pain was due to a small hernial protrusion of the subperitoneal fat through a small hole in the abdominal aponeurosis in the linea alba above the umbilicus. The removal of the fatty outgrowth cured the patient. In some cases the abdominal pain had been treated by almost every drug employed for the cure of indigestion. The pain usually came on after any sudden exertion or after taking a large meal. Mr. Bennett also points out that pain referred to the umbilicus or the loin may be due to the slipping up of the testicle into the inguinal canal. This condition is, of course, due to a failure in the obliteration of the upper portion of the tunica vaginalis, and is best treated by the application of a well-fitting light truss.

**Vesical Hernia.**—The subject of vesical hernia has lately received considerable attention. Dr. Ernst Michels communicated to the Medico-Chirurgical Society[2] a case in which during the operation for radical cure of hernia the bladder was found and a portion removed in mistake for the sac. The stump was put back into the abdomen and the inguinal canal closed. The mistake was not discovered until twenty-four hours after the operation, when the patient began to complain of pain in the hypogastrium and to pass blood with the urine. The bladder was fully exposed by abdominal section, and the wound, which was discovered in its extra peritoneal part, closed with sutures, the bladder being drained by a Jacques' catheter. The author of the communication pointed out that there were two distinct kinds of vesical hernia; in one the bladder was contained within a peritoneal sac, but in the other the extra peritoneal portion of the bladder descended, and this was a much rarer condition. The bladder had been wounded in almost all the cases the records of which the author had examined, as it was exceedingly difficult to recognise it. With this Mr. Macready agreed, and stated that the accidental opening of the bladder in these cases had been done by many accomplished surgeons, and that he had himself done so. In his case the wound of the bladder was not discovered until the ninth day, when urine was found trickling from the wound. Mr. Arbuthnot Lane[3] records a case in which a hernial protrusion of the bladder was found outside the true sac in the operation for the cure of a large inguinal hernia. On pressure fluid could be forced out of it into the abdomen. It was thought to be a portion of the bladder, but to make sure was opened and then sutured. It was freed from its adhesions to the outside of the hernial sac, and returned into the abdomen. A case in which a portion of the bladder formed part of a hernia is reported by Mr. Rose,[4] which differs from the other recorded cases in that symptoms of bladder trouble were present. Micturition was very painful at times, and there was retention occasionally, associated with some increase in the size of the hernia. The radical cure operation was performed, and the painful micturition ceased. The hernia was an inguinal one, although the patient was a young woman. The cases of Mr. Lane and Mr. Rose are, however, quite different from Dr. Michels' case, in which the bladder alone protruded, and resembled the hernial sac itself, a condition in which the bladder has almost always been taken for the sac, and opened as such, happily without any serious consequences, the opening thus accidentally made having been carefully sutured in each case.

**Interstitial Hernia.**—A case of this nature, associated with malposition of the testes, is recorded by Mr. Francis.[5] The hernial sac formed a double pouch, one portion lying in the aponeurosis of the external oblique muscle, and the other beneath it. The sac failed to reach the scrotum because of imperfectly developed condition of the latter structure, due to the retention of the testicle in the inguinal canal. The author points out that in interstitial hernia the sac has been often described as lying on the aponeurosis of the external oblique, seldom between it and the internal oblique, but very rarely indeed have two distinct pouches been recorded, one above and one below the aponeurosis.

**Radical Cure of Hernia.**—Dr. Phelps[6] describes a new method of performing radical cure, or, rather, modifies the existing open methods in some particulars. He objects to the use of catgut or other absorbable materials in closing the abdominal parietes; but the only really novel point about his operation is the way in which he puckers together and inverts the neck of the sac, and then by special suturing closes the internal abdominal ring. The inguinal canal is entirely obliterated by being stitched up with fine silver wire, and the cord is brought out through the different layers of muscles at a different point, thus making one layer of muscles act as a guard against the opening in the layer underneath.

**Inflamed and Irreducible Congenital Umbilical Hernia.**—A congenital umbilical hernia is always a serious thing, as the separation of the dead portion of cord on its surface is very likely to set up septic inflammatory mischief in the rupture. Such a condition is recorded by Mr. D'Arcy Power, from the Victoria Hospital for Sick Children, Chelsea.[7] The infant was two days old, and had vomited twice on the day of admission, but the bowels had acted several times since birth. The hernia was not strangulated, but the intestine in the sac was matted together with a thick layer of lymph. It consisted of the cæcum with the vermiform appendix and portions of the ileum and colon. The adhesions were separated and the hernial contents returned into the peritoneal cavity, but the child only lived 24 hours, slight general peritonitis being found at the post-mortem.

[1] Lancet, Sept. 15, 1894, p. 617. [2] Lancet, 1894, vol. i., p. 1C68. [3] Ibid, 1894, vol. i., p. 1186. [4] Ibid, 1894, vol. ii., p. 194. [5] The Quarterly Med. Journal, April, 1894, p. 217. [6] The New York Med. Journal, Sept. 8, 1894, p. 291. [7] Lancet, 1894, Nov. 24.

